# High-Resolution 3D Structure Determination of Kaliotoxin by Solid-State NMR Spectroscopy

**DOI:** 10.1371/journal.pone.0002359

**Published:** 2008-06-04

**Authors:** Jegannath Korukottu, Robert Schneider, Vinesh Vijayan, Adam Lange, Olaf Pongs, Stefan Becker, Marc Baldus, Markus Zweckstetter

**Affiliations:** 1 Department for NMR-based Structural Biology, Max Planck Institute for Biophysical Chemistry, Göttingen, Germany; 2 Zentrum für Molekulare Neurobiologie, Institut für Neurale Signalverarbeitung, Hamburg, Germany; The University of Manchester, United Kingdom

## Abstract

High-resolution solid-state NMR spectroscopy can provide structural information of proteins that cannot be studied by X-ray crystallography or solution NMR spectroscopy. Here we demonstrate that it is possible to determine a protein structure by solid-state NMR to a resolution comparable to that by solution NMR. Using an iterative assignment and structure calculation protocol, a large number of distance restraints was extracted from ^1^H/^1^H mixing experiments recorded on a single uniformly labeled sample under magic angle spinning conditions. The calculated structure has a coordinate precision of 0.6 Å and 1.3 Å for the backbone and side chain heavy atoms, respectively, and deviates from the structure observed in solution. The approach is expected to be applicable to larger systems enabling the determination of high-resolution structures of amyloid or membrane proteins.

## Introduction

Structural characterization of membrane proteins and many other biological systems by X-ray crystallography or solution NMR spectroscopy is difficult because of problems with crystallization, solubility or molecular size. Significant advances, however, have been made to construct three-dimensional (3D) molecular structures from solid-state NMR data obtained under Magic Angle Spinning (MAS)[1] conditions[2], [3], [4]. These efforts resulted in high-resolution 3D conformations for small peptides[5], [6], [7], [8] and the determination of medium-resolution backbone structures for a few solid-phase proteins.[9], [10], [11], [12].

Structure determination from solid-state NMR data typically follows the approach employed by solution-state NMR, namely assignment of backbone and side chain resonances using pulse sequences for sequential correlation of resonances, characterization of torsion angles and detection of tertiary contacts. Unless sample orientation provides a direct route to monitor molecular structure under MAS conditions[13], [14], the collection of medium and long-range distance constraints is most crucial. Ideally, these correlations are closely related to molecular structure, can be measured in high spectral resolution and lead to unequivocal assignments of structure-relevant correlations. Two strategies have been developed in this direction: (i) measurement of ^13^C-^13^C distances on ^13^C block-labeled protein microcrystals[9] and (ii) extraction of ^1^H-^1^H-distance restraints from ^13^C,^13^C- and ^15^N,^13^C-encoded ^1^H/^1^H mixing experiments on a uniformly ^13^C/^15^N-labeled sample[11].

Here we combine ^13^C,^13^C- and ^15^N,^13^C-encoded ^1^H/^1^H mixing experiments recorded on a uniformly ^13^C/^15^N-labeled sample with a probabilistic assignment algorithm originally developed for the automatic assignment of ^1^H-^1^H correlations in Nuclear Overhauser Effect spectra recorded on proteins in solution[15]. We determine the high-resolution structure of the 38-residue scorpion toxin kaliotoxin (KTX) and show that the structure of KTX in the solid phase deviates from the one observed in solution.

## Results and Discussion

Earlier, the backbone fold of the 38-residue potassium channel blocker toxin KTX in the solid phase was deduced from 28 manually assigned interresidue CHHC correlations (ProteinDataBank (PDB) code: 1XSW)[11]. To define the structure of KTX at higher accuracy, a significantly higher number of medium and long-range correlations was required. For this aim, we set out to combine analysis tools originally developed for the assignment of internuclear correlations in liquid-state NMR spectra with solid-state NMR data. To avoid intermolecular contacts, the ^13^C/^15^N-labeled protein was diluted six-fold (when compared to previous measurements [11]) by the addition of unlabeled KTX. In addition, mixing times in 2D CHHC experiments were reduced to 100, 175 and 250 µs to reduce the potential impact of spin diffusion. The three 2D CHHC spectra and one 2D NHHC spectrum were analyzed using PASD, a probabilistic assignment algorithm for automated structure determination [15]. Three successive PASD passes of cross peak assignment and simulated annealing were performed and each pass was started from a set of randomly generated coordinates. Calculations were carried out in torsion angle space using assigned distance restraints along with torsion angle restraints predicted from backbone chemical shifts using the program TALOS[16], [17] [18]. After completion of the PASD calculations, cross peak assignments were selected that had a good fit to the 1XSW backbone fold (PASD assignment likelihood of 1.0). Subsequently, a high-resolution structure was calculated on the basis of selected cross peak assignments using an optimized simulated annealing protocol.[19] These calculations were started from random initial coordinates, all verified distance restraints were active during the course of calculation and torsion angle restraints predicted by TALOS were included.

**Figure 1:**
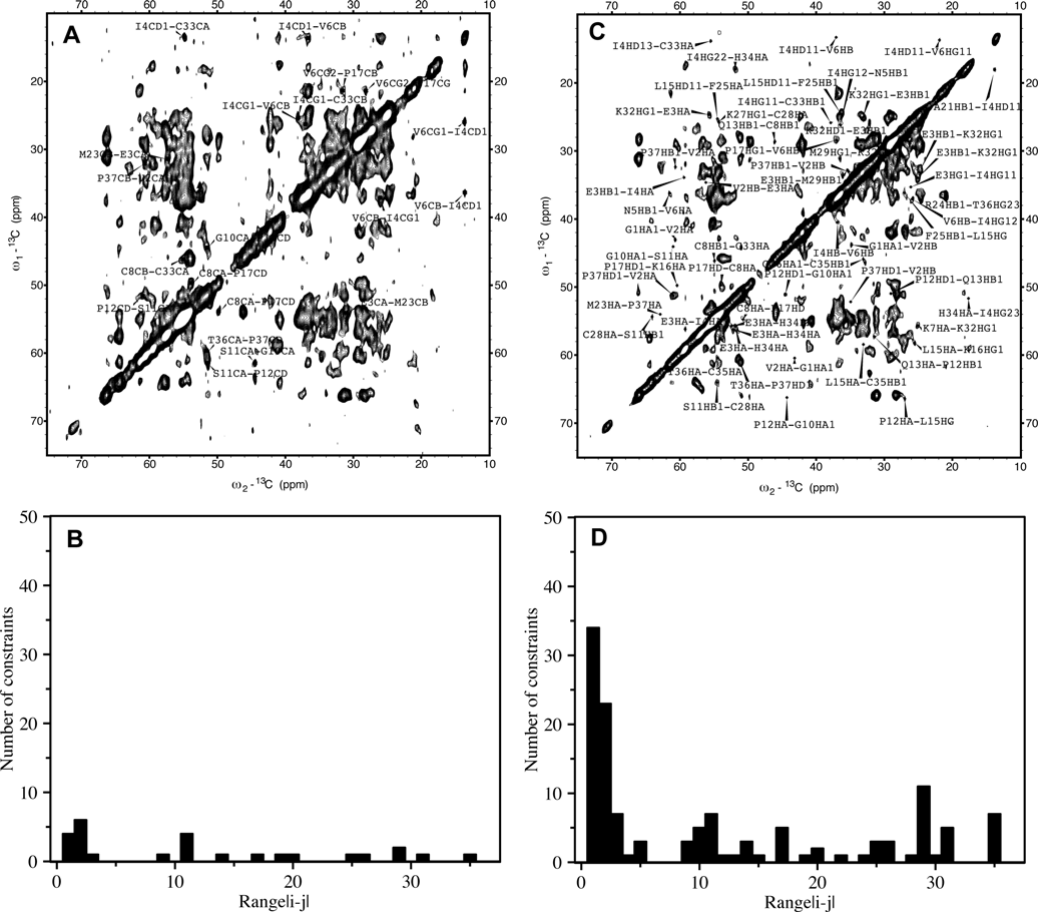
Comparison of interresidue correlations assigned earlier [11] (A and B) and assigned in this study (C and D) for KTX in the solid phase. Signals assigned in the 2D CHHC spectrum of diluted U-[^13^C, ^15^N]-KTX recorded with a mixing time of 250 µs are labeled. (B) and (D) show the number of unambiguously assigned distance constraints as a function of residue difference i and j.

**Figure 2:**
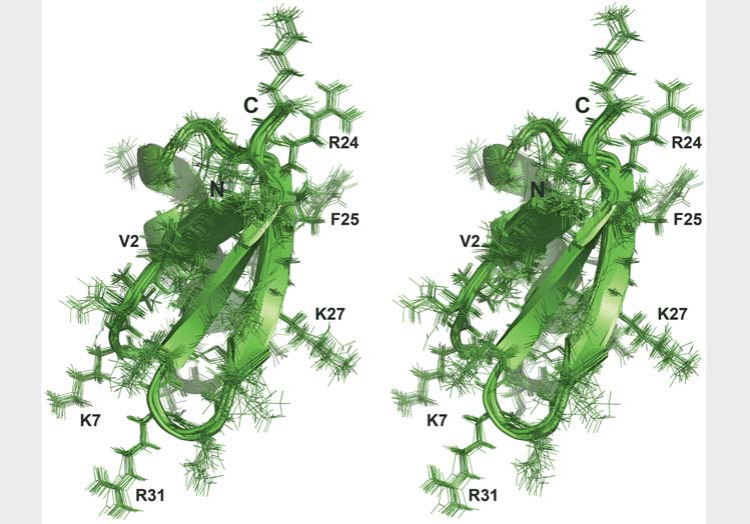
High-resolution 3D structure of KTX determined in the solid phase. Stereo view of the 20 lowest-energy structures are shown.

**Figure 3:**
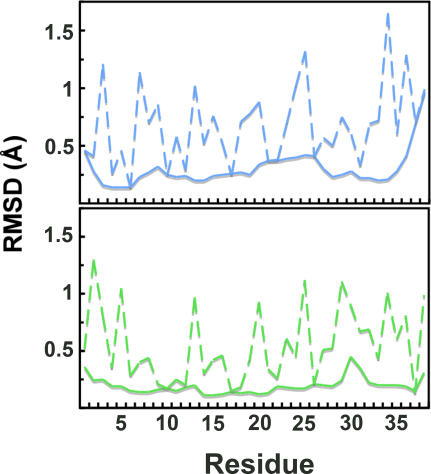
Coordinate precision of KTX in solution (A) and solid phase (B). Shown are residue-based rms deviations of the coordinates of backbone atoms (solid line) and non-hydrogen side chain atoms (dashed line) within the ensemble of 20 lowest energy structures.

Previously, 15 long-range, 7 medium-range and 6 short-range correlations could be assigned [11]. Using the above described semi-automated approach a total of 260 ^1^H-^1^H distance correlations could be assigned unambiguously (Figure 1CD and Table 1). 62 of these were long-range, 33 medium-range and 165 sequential. The 3D solid-state structure of KTX that was calculated from the 260 distance restraints and 58 dihedral angle restraints is shown in Figure 2. The resulting ensemble of KTX structures tightly converged with a coordinate precision of 0.6 Å and 1.3 Å for backbone and side chain heavy atoms, respectively. Backbone and most side chains had a well-defined orientation except the N- and C- terminal residues and Asn30 located in the loop connecting the second and third β-strand of KTX (Figure 3). The high-resolution solid-state structure of KTX deviates by 2.4 Å from the backbone conformation (PDB code: 1XSW) obtained on the basis of 28 manually assigned distance restraints[11], which deviates by 2.7 Å from the solution structure. The most pronounced deviation between 1XSW and the high-resolution structure was observed at the N-terminus, where four residues were rotated by about 50°, such that the first β-strand was straight and not bent as seen in the high-resolution structures (Figure S1).

**Table 1:** Structural statistics for the 20 lowest-energy structures of KTX in solution and in the solid phase.

	Solution	Free
**Proton-Proton distance correlations**		
Total	314	260
Short range	199	165
Medium range	45	33
Long range	70	62
Distance violations (>0.5 Å)	0	2^b^
**Dihedral angles**	64	58
Dihedral violations (>5°)	0	0
**Energy(kcal/mol)**		
Total	−1203.3±60.1	−1365.1±62.3
Dihedral	−133.4±5.6	−119.3±5.1
NOE/CHHC	−166.8±34.3	−167.7±29.3
**RMSD**		
Dihedral	3.2±1.3	4.9±1.0
NOE/CHHC	0.08±0.02	0.09±0.01
**Coordinate precision ** a		
Backbone atoms (Å)	0.7	0.6
All heavy atoms (Å)	1.6	1.3
**Ramachandran statistics**		
Most favored region (%)	86.7	84.0
Disallowed region (%)	3.3	3.7

a Defined as the average rmsd difference between the 20 structures and the mean coordinates.

b The two distance restraints G1(Hα1)-I4(Hα) and I4(Hγ11)-C35(Hα) were violated by 0.69 and 0.6 Å, respectively. The two restraints came from weak cross peak in the spectra and were assigned a distance range of 1.8–6.0 Å in the calculations.

Various tests were performed to probe the convergence of the structure calculations and support the accuracy of the high-resolution solid-state structure (see Materials and Methods): (i) use of CHHC spectra with longer mixing times and at six-fold higher concentration; (ii) use of different conformations for calculating likelihood estimates in PASD; (iii) influence of chemical shift tolerances; (iv) sensitivity towards distance ranges used for interresidue correlations; (v) dependence on the number of CHHC spectra; (vi) influence of disulphide bond restraints. In all cases, the backbone of the calculated structures deviated by less than 0.7 Å from the backbone of the structure shown in Figure 2.

Recently, a method for automatic assignment of cross peaks in ^13^C-^13^C correlation spectra was developed[20]. The approach called SOLARIA was used to analyze proton-driven spin diffusion spectra recorded on ^13^C– block-labeled, microcrystalline preparations of the α-spectrin SH3 domain. In this study, only a modest improvement in the 3D backbone structure was observed. In contrast, our strategy based on C/NHHC correlations leads to an atomic resolution definition of both the backbone and the side-chain structure of KTX. We attribute these improvements to the higher fraction of long-range contacts in initial-rate N/CHHC spectra that allows for the same small distances boundaries[21], [22] during structure calculation as used in liquid-state NMR.

To enable a direct comparison, we determined the solution structure of KTX employing the identical strategy as used for KTX in the solid phase. 70 long-range, 45 medium-range and 199 sequential NOEs could be assigned unambiguously, closely resembling the amount and distribution of distance restraints obtained from 2D N/CHHC spectra for KTX in the solid phase (Table 1). The newly determined solution structure deviates by 0.6 Å from a previously determined solution structure of KTX (PDB code: 2KTX)[23].

**Figure 4:**
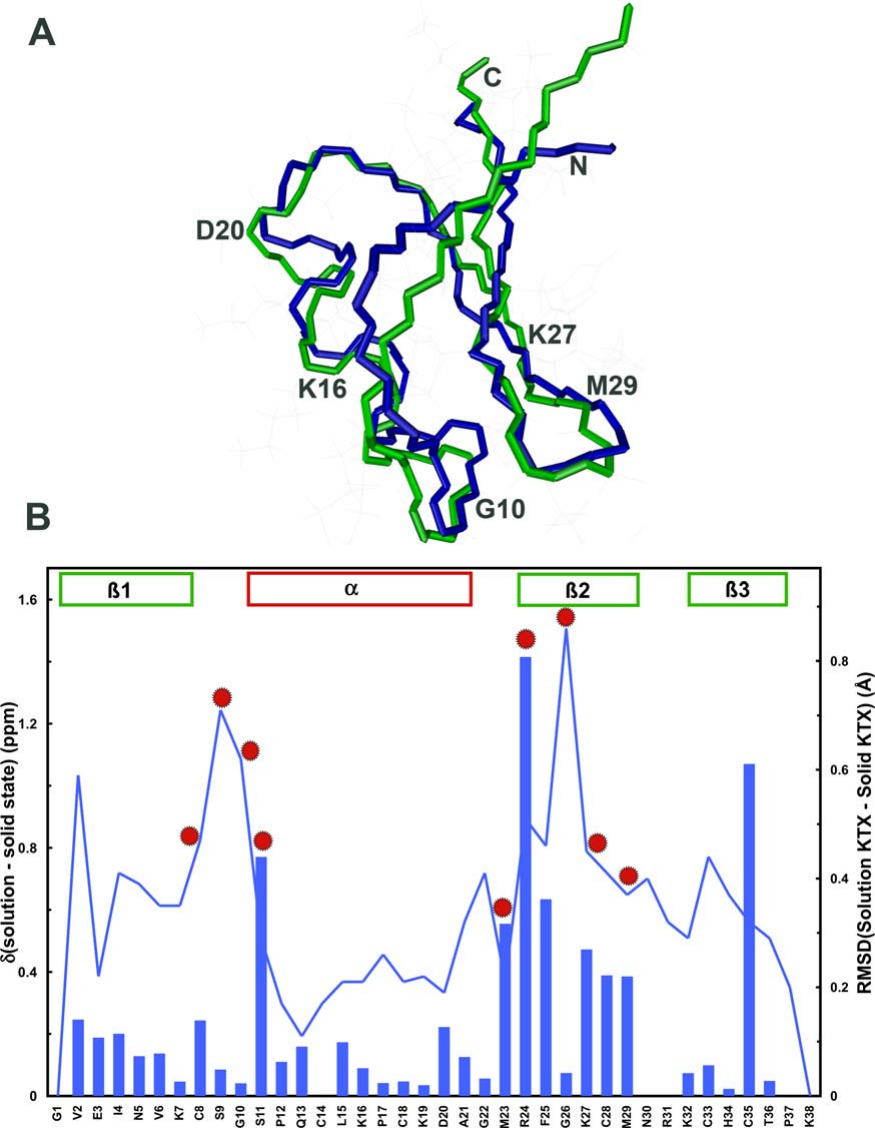
(A) Superposition of high-resolution solid-state structure (green) and solution structure (blue; PDB code: 2KTX) of KTX. (B) Comparison of averaged Cα/Cβ chemical shift differences (blue bars; calculated according to 0.256*[ΔδCα^2^+ΔδCβ^2^]^1/2^) with rms deviation between the mean structures (blue line) of KTX in solution and in the solid-phase. Red dots mark residues, for which the backbone dihedral angles predicted by TALOS differ in solution and the solid state. Secondary structure is indicated.

The backbone of the high-resolution solid-state structure of KTX deviates by 1.3 Å from that observed in solution (Figure 4A). Structural differences were observed for the two N-terminal residues, the loop between the first β-strand and the α-helix, and the C-terminal β-sheet in particular next to G26 (Figure 4B). The structural differences are due to a combination of changes in interresidue cross peaks (Table S1) and in backbone dihedral angles. For residues 8–11, 23–25, K27 and M29, backbone dihedral angles predicted by TALOS from the solid-state chemical shifts clearly deviated from those predicted by TALOS from the solution-state chemical shifts (Figure 4B and Figure S2).

### Conclusion

Our study demonstrates that high-resolution 3D structures of globular proteins can be obtained from solid-state NMR data. The combination of ^15^N,^13^C-encoded ^1^H/^1^H mixing experiments with a probabilistic cross peak assignment algorithm is particularly powerful, as short distances between protons provide the principal source of long-range structural information. Depending on the molecule under investigation, the presented approach can be combined with other solid-state NMR spectroscopic methods. Applications to larger proteins may benefit from the use of block[9], modular[24] or stereo-array[25] isotope labeling, and allow the determination of high-resolution structures of amyloid or membrane proteins.

## Materials and Methods

### NMR spectroscopy

Unlabelled and uniformly [^13^C,^15^N]-labeled KTX was prepared recombinantly as previously described[11], [34].

Solution-state NMR experiments were carried out at 298 K on a Bruker AVANCE 600 spectrometer. NMR samples contained 2 mM unlabelled KTX in 95% H_2_O/5% D_2_O, pH 7.5. 2D TOCSY (mixing time of 60 ms using MLEV17) and NOESY experiments (mixing time of 200 ms) were used to facilitate backbone assignment[26], [27]. The spectra were recorded using 362×724 complex data points in F_1_ and F_2_ dimensions with 32 scans per increment and a relaxation delay of 1.2 s. The spectral widths were 9615 and 9603 Hz in the F1 and F2 dimensions, respectively. ^13^C^α^, ^13^C^β^ and ^15^N chemical shifts were obtained from natural abundance, two-dimensional ^1^H-^15^N and ^1^H-^13^C HSQCs[28]. All data were processed using NMRPipe[29].

An anisotropic medium for measurement of residual dipolar couplings was prepared by addition of Pf1 filamentous phages (Asla, Riga, Latvia) to a concentration of 12 mg/ml[30]. To lower the electrostatic attraction between KTX and the highly negatively charged Pf1 phage, the ionic strength was raised to 500 mM. ^1^H^N^-^15^N splittings were measured under isotropic and anisotropic conditions using 2D IPAP-^1^H-^15^N HSQC experiments[31]. RDCs were extracted by subtraction of the ^1^ *J*_NH_ scalar coupling measured for the isotropic sample. Comparison of experimental RDCs (^1^ *D*_NH_) with values back-calculated from the redetermined solution-state structure of KTX using singular value decomposition as implemented in PALES[32] resulted in a Pearson's correlation coefficient of 0.93. The magnitude *D*_a_ and rhombicity *R* of the alignment tensor were back-calculated as *D*_a_ = 2.5 Hz and *R* = 0.23. When best-fitting the experimental RDCs to the published solution-state structure (PDB code: 2KTX), a Pearson's correlation coefficient of 0.85 was obtained.

Solid-state NMR data comprised three CHHC spectra (100, 175 and 250 µs (^1^H,^1^H) mixing) and one NHHC spectrum (100 µs (^1^H,^1^H) mixing) [11]. CHHC spectra were obtained on a uniformly [^13^C,^15^N]-labeled KTX sample diluted approximately 1∶6 in unlabeled KTX, while the NHHC spectrum was recorded on an undiluted uniformly [^13^C,^15^N]-labeled sample. Data were recorded on a wide-bore Bruker 600 MHz instrument at 11 kHz MAS speed (CHHC spectra with 100 and 175 µs mixing time) and on a standard-bore Bruker 800 MHz instrument at 12.5 kHz MAS (CHHC spectrum with 250 µs mixing time and NHHC spectrum) using 4 mm triple-resonance (^1^H,^13^C,^15^N) probes. Sample temperature was about 280 K in all cases. ^1^H field strengths used for 90° pulses and SPINAL64 [33] decoupling during evolution and detection periods were between 70 and 83 kHz. Short CP contact times of t_HC_ = 100 µs or t_HN_ = 250 µs enclosing the (^1^H,^1^H) transfer step were employed to ensure polarization transfer between directly bonded nuclei only. Spectra were recorded with 105×1024 (CHHC 100 and 175 µs), 140×1280 (CHHC 250 µs) or 40×1536 (NHHC) complex data points in F1 and F2 dimensions, respectively, with around 1024 (CHHC) or 2048 (NHHC) scans per increment. Spectral widths were 83 (CHHC) or 44 (NHHC) ppm in the indirect dimension and 310 to 355 ppm in the direct dimension, respectively. The recycle delay was set to 2s.

### Automated cross-peak assignment and structure calculation

Two-dimensional CHHC, NHHC and NOESY spectra were automatically peak picked using Sparky 3 (T. D. Goddard and D. G. Kneller, University of California, San Francisco). Diagonal peaks were manually removed. Peak intensities were classified into four ranges and converted into distance ranges of 1.8–2.7, 1.8–3.3, 1.8–5.0, and 1.8–6.0 Å. Lists of cross peaks were subjected to the automated cross-peak assignment and structure calculation algorithm PASD implemented in Xplor-NIH[15], [17]. For analysis of the solid-state spectra by PASD, ^13^C and ^15^N chemical shifts were labeled as if they were proton chemical shifts. Tolerances for matching chemical shifts to cross-peaks were 0.015 ppm in F2 and F1 for the NOESY spectrum, and 0.38 ppm and 0.60 ppm in the acquisition and indirect dimension of the N/CHHC spectra, respectively.

PASD was applied largely following published procedures[15]. In short, three successive passes of simulated annealing calculations in torsion angle space were carried out. Each pass was started from a set of randomly generated coordinates. The target function comprised a potential function for experimental distance restraints (e.g. obtained from NOEs or CHHC correlations), a quadratic van der Waals repulsion term, a square-well potential for torsion angles and a torsion angle database potential of mean force. Pass 1 and 2 protocol comprised two high-temperature phases (4000 K) and a slow cooling phase (from 4000 to 100 K) with a linear NOE potential. Pass 3 comprised a single high-temperature phase (4000 K) followed by a cooling phase with a quadratic NOE potential. Final assignment likelihoods were determined at the end of pass 3 calculations. Calculations were carried out on a Linux cluster of 32 processors and took about two days for each structure.

PASD structures do not represent fully-refined NMR structures[15]. Therefore, we selected cross-peaks that were in agreement with the backbone fold of KTX determined previously (see below): for KTX(solution) and KTX(solid), 31% and 28%, respectively, of all long-range restraints, 83% and 80%, respectively, of the medium-range restraints, and 99% and 100%, respectively, of the sequential restraints had final restraint likelihoods of 1.0. Assignments obtained for these cross-peaks by PASD were verified by manual inspection of the 2D N/CHHC spectra for KTX(solid) or the NOESY spectrum for KTX(solution).

### Convergence of automated cross-peak assignment and structure calculation

We performed several tests to probe the reliability of the solid-state 3D structure of KTX: (i) use of CHHC spectra recorded with longer mixing times and using undiluted ^13^C/^15^N-labeld KTX; (ii) use of different conformations for calculating likelihood estimates in the PASD analysis; (iii) influence of chemical shift tolerances; (iv) influence of distance ranges; (v) influence of disulphide bond restraints; (vi) combination of solid-state distance restraints with solution-state dihedral angles (and vice versa).

To i): In contrast to the measurements performed in this study, CHHC spectra were previously recorded on ^13^C/^15^N-labeld KTX that was not diluted by unlabeled protein [11]. In these spectra, intermolecular cross peaks may appear. In addition, the spectra had been recorded with mixing times of 250, 325 and 400 µs, increasing the risk of spin diffusion. Nevertheless, when using these three CHHC spectra together with the 2D NHHC spectrum, the resulting structure deviated by less than 0.7 Å (rms value for all N, Cα, CO backbone atoms) from the structure shown in Figure 2.

To ii): At the end of pass 1 and 2 the PASD algorithm calculates likelihood estimates that each particular assignment associated with a cross-peak is correct. The likelihoods are calculated using the ensemble of structures present at the end of the corresponding pass. Thus, they are a metric of how consistent a given assignment is with the ensemble of structures at the end of each calculation pass[15]. Here we have not used the ensemble of structures present at the end of pass 1 and 2 for calculation of likelihood estimates, but either the high-resolution structure of KTX obtained under different conditions or a medium-resolution backbone fold. This improved convergence in the structure calculations and was justified as we previously established that the fold of KTX in solution and in the solid phase is the same[11]. The PASD calculations of KTX(solid) were done once by using KTX(solution) (PDB CODE: 2KTX) for calculation of the likelihood estimates at the end of pass 1 and 2. Thus, we biased on purpose the calculation towards the solution-state structure. Then a second PASD calculation was done, in which the likelihood estimates were determined using the medium resolution backbone fold obtained previously for KTX in the solid phase (PDB CODE: 1XSW).[11] In all cases, the structures obtained from the two different PASD calculations were indistinguishable. This supports the relevance of the differences between the solution and solid-state structure. Note, that identical structure calculation protocols were used in all cases.

To iii): For the calculations reported in the main part of the manuscript, tolerances for matching chemical shifts to cross-peaks were set to 0.38 ppm and 0.60 ppm in the acquisition and indirect dimension, respectively. We repeated the structure calculations with chemical shift tolerances of 0.38 ppm and 0.4 ppm in the acquisition and indirect dimension, respectively. The resulting structure deviated by less than 0.7 Å (rms value for all N, Cα, CO backbone atoms) from the structure shown in Figure 2.

To iv): Peak intensities obtained from the 2D CHHC and NHHC spectra were classified into four ranges and converted into distance ranges of 1.8–2.7, 1.8–3.3, 1.8–5.0 and 1.8–6.0 Å, respectively. The classification was done independently for the four proton-proton correlation spectra (see main manuscript). To test the sensitivity of the solid-state structure to the used distance ranges, we repeated the structure calculations assigning to all N/CHHC correlations a distance range of 2.4–6.0 Å. The resulting structure deviated by less than 0.3 Å (rms value for all N, Cα, CO backbone atoms) from the structure shown in Figure 2.

To v): For both KTX(solution) and KTX(solid), structure calculations were performed without and with restraints for the three disulphide bonds. The resulting structures did not differ (backbone rms deviation below 0.5 Å) and only the results of calculations, in which the disulphide bonds were not enforced, were reported.

To vi): Are the structural differences due to an uncertainty in the analysis of N/CHHC spectra? To address this question, we recalculated the structure (using XPLOR-NIH and starting from an extended strand) using the same solid-state N/CHHC distance restraints, but supplementing them with the dihedral angles obtained by TALOS from the solution-state chemical shifts (instead of those obtained from the solid-state chemical shifts). The backbone of the resulting structure deviated by 0.5 Å from the high-resolution solid-state structure. The coordinate precision for backbone and all heavy atoms was 0.7 Å and 1.7 Å, respectively. However, two dihedral angle violations were introduced (for residues 2 and 24) and residue 24 moved into the disallowed region of the Ramachandran plot. In addition, the total energy increased from −1307±54 kcal/mol to −1032±48 kcal/mol, the dihedral angle energy from −110±6 kcal/mol to −16±36 kcal/mol and the distance restraint energy from −157±28 kcal/mol to −18±67 kcal/mol (when compared to the pure solid-state structure calculation). Similarly, when the solution-state distance restraints were combined with the solid-state dihedral angles, one dihedral angle violation (for S9) was introduced, the total energy was increased from 1203±60 kcal/mol to −1154±65 kcal/mol, the dihedral angle energy from −133±6 kcal/mol to −55±32 kcal/mol and the distance restraint energy from −167±34 kcal/mol to −31±53 kcal/mol (when compared to the pure solution-state structure calculation). The backbone of the resulting structure deviated by 0.6 Å from the high-resolution solution-state structure. The coordinate precision for backbone and all heavy atoms was 0.8 Å and 1.9 Å, respectively. These data demonstrate that the solid-state distance restraints are only in agreement with the solid-state backbone chemical shifts, and the solution-state distance restraints are only in agreement with the solution-state backbone chemical shifts.

## Supporting Information

Table S1 (0.50 MB DOC)

Figure S1 (0.21 MB DOC)

Figure S2 (0.10 MB DOC)
