# Supplementary material for: High-Resolution 3D Structure Determination of Kaliotoxin by Solid-State NMR Spectroscopy
Source: PLoS One. 2008 Jun 4;3(6):e2359. doi: 10.1371/journal.pone.0002359 (PMC2387072; doi:10.1371/journal.pone.0002359)

**Table S1.** Selected cross peaks in solid-state CHHC spectra of KTX that are not visible in the solution-state NOESY spectrum.

| **Cross peak** | **Distance in the solution state structure [Å]** | **Distance in the solid state structure [Å]** |
| --- | --- | --- |
| 4 HD1 - 21 HB | 7.97 | 5.32 |
| 8 HB1 - 13 HA | 8.28 | 6.38 |
| 8 HB1 - 13 HB1 | 6.54 | 4.49 |
| 8 HA - 17 HD ^a^ | 8.36 | 5.80 |

^a^ Below, part of the solid-state CHHC spectrum (mixing time = 250 μs) is shown in green, in which the C8(Hα)-P17(HD) cross peak was found. Note that the peak is present on both sides of the diagonal. In the solution-state NOESY spectrum (mixing time = 200ms, shown in red), no cross peak could be detected at the chemical shifts of C8(Hα) and P17(HD) (marked by a double cross).


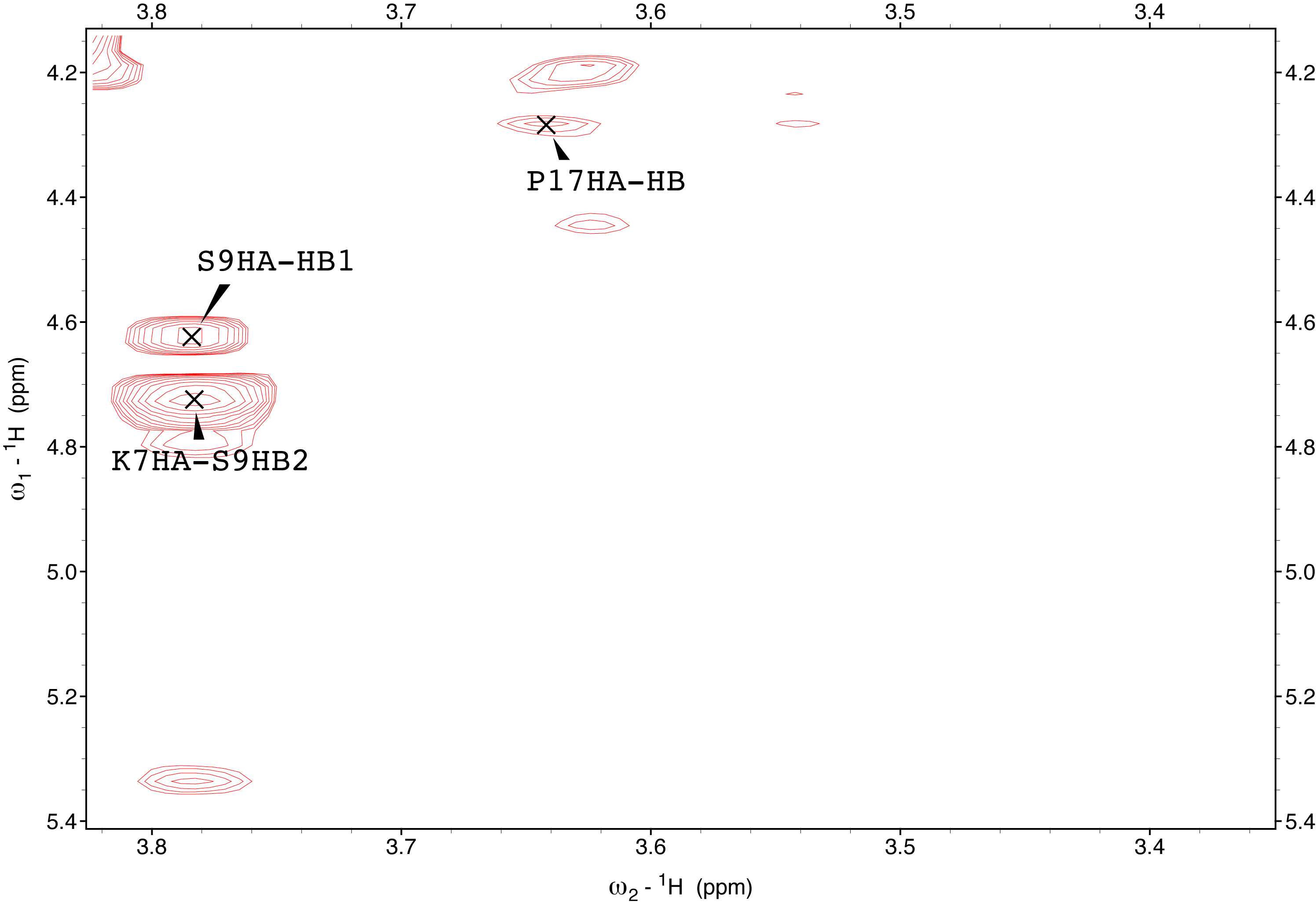


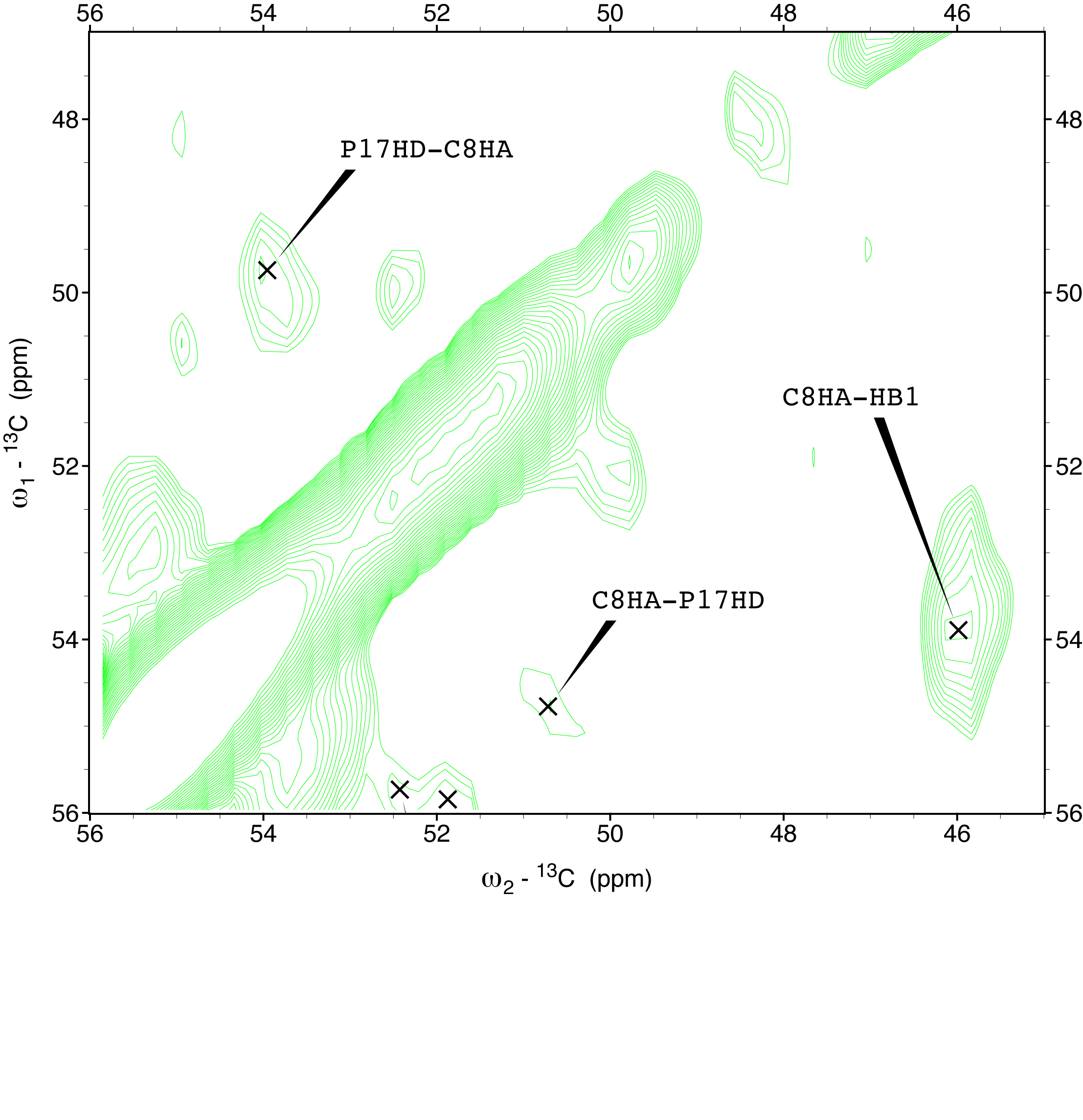

Supplement: Table S1 — (0.50 MB DOC) [file pone.0002359.s001.doc]
