# Supplementary material for: High-Resolution 3D Structure Determination of Kaliotoxin by Solid-State NMR Spectroscopy
Source: PLoS One. 2008 Jun 4;3(6):e2359. doi: 10.1371/journal.pone.0002359 (PMC2387072; doi:10.1371/journal.pone.0002359)
Supplement: Figure S1 — (0.21 MB DOC) [file pone.0002359.s002.doc]

**
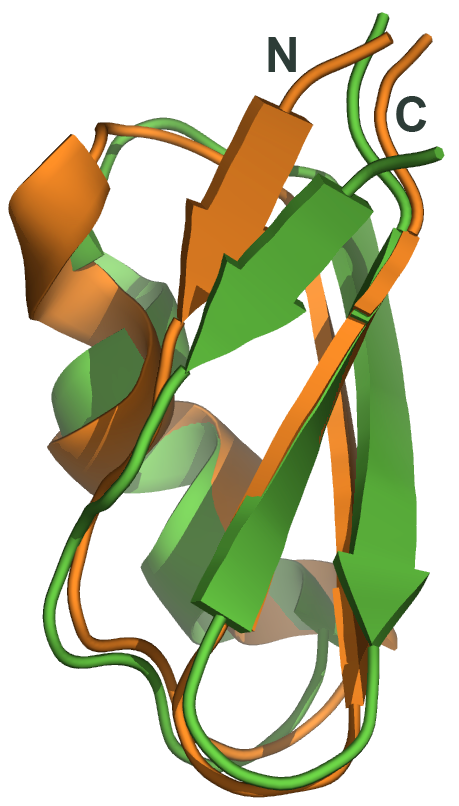
**

**Figure S1** High-resolution solid-state structure of KTX (green; determined in this study) compared to the backbone fold obtained previously from 28 manually assigned CHHC correlations (orange; PDB code: 1XSW).
