# Supplementary material for: High-Resolution 3D Structure Determination of Kaliotoxin by Solid-State NMR Spectroscopy
Source: PLoS One. 2008 Jun 4;3(6):e2359. doi: 10.1371/journal.pone.0002359 (PMC2387072; doi:10.1371/journal.pone.0002359)
Supplement: Figure S2 — (0.10 MB DOC) [file pone.0002359.s003.doc]

**
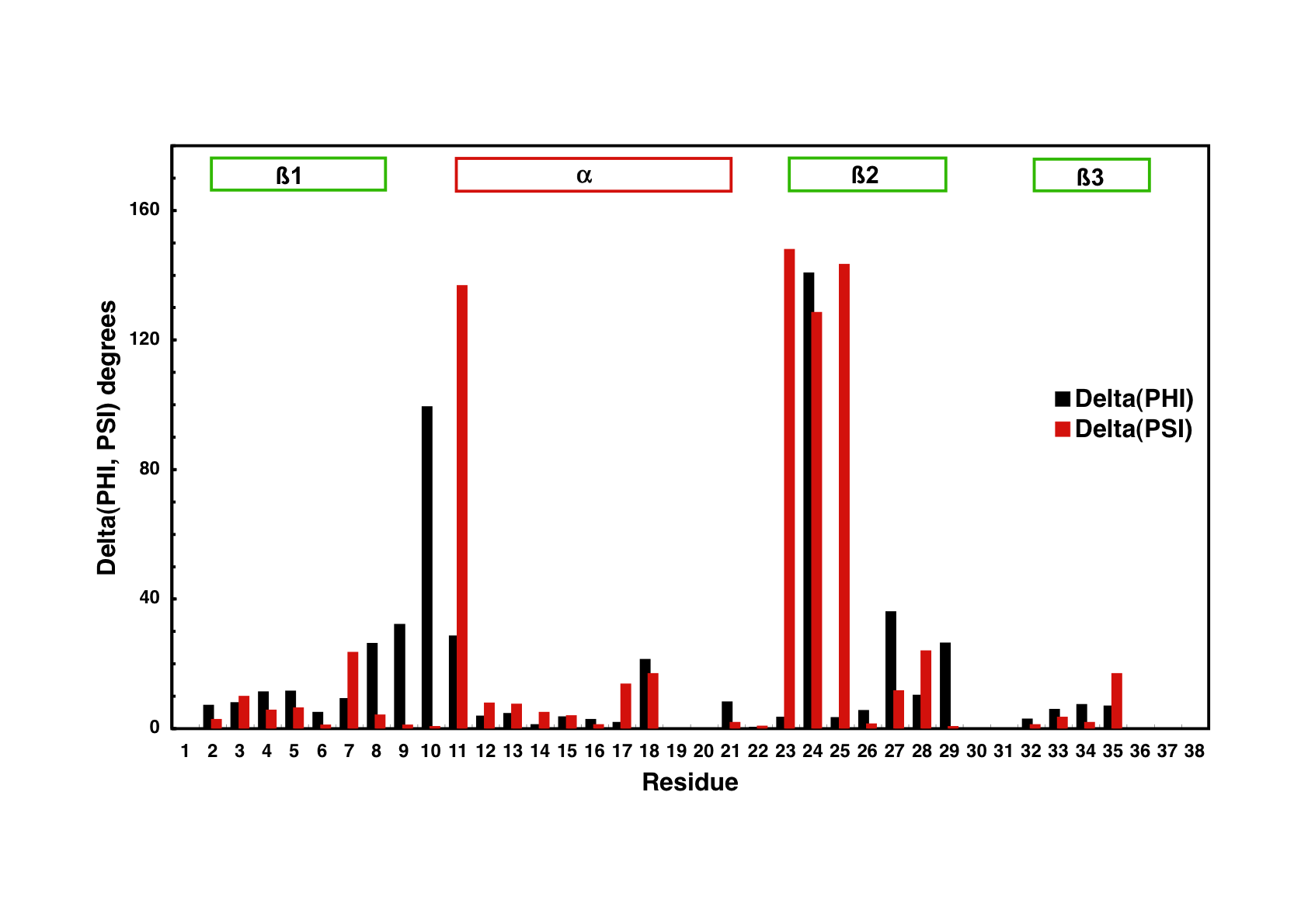
**

**Figure S2** Differences between the backbone dihedral angles predicted by TALOS from the solution-state and the solid-state chemical shifts. The average uncertainty of TALOS predictions as stated on the web page is ~12°. In Figure 2b, residues were marked with a red dot, for which the difference in either *phi* or *psi* exceeds twice this value.
